# The discovery of a new antibody for BRIL-fused GPCR structure determination

**DOI:** 10.1038/s41598-020-68355-x

**Published:** 2020-07-15

**Authors:** Hikaru Miyagi, Hidetsugu Asada, Michihiko Suzuki, Yuichi Takahashi, Mai Yasunaga, Chiyo Suno, So Iwata, Jun-ichi Saito

**Affiliations:** 1 R&D Division, Kyowa Kirin Co., Ltd., Tokyo, Japan; 2 Department of Cell Biology, Graduate School of Medicine, Kyoto University, Kyoto, Japan; 3 R&D Division, Kyowa Kirin Co., Ltd., Shizuoka, Japan; 4 RIKEN, SPring-8 Center, Hyogo, Japan

**Keywords:** Pharmaceutics, X-ray crystallography

## Abstract

G-protein-coupled receptors (GPCRs)—the largest family of cell-surface membrane proteins—mediate the intracellular signal transduction of many external ligands. Thus, GPCRs have become important drug targets. X-ray crystal structures of GPCRs are very useful for structure-based drug design (SBDD). Herein, we produced a new antibody (SRP2070) targeting the thermostabilised apocytochrome b562 from *Escherichia coli* M7W/H102I/R106L (BRIL). We found that a fragment of this antibody (SRP2070Fab) facilitated the crystallisation of the BRIL-tagged, ligand bound GPCRs, 5HT_1B_ and AT_2_R. Furthermore, the electron densities of the ligands were resolved, suggesting that SPR2070Fab is versatile and adaptable for GPCR SBDD. We anticipate that this new tool will significantly accelerate structure determination of other GPCRs and the design of small molecular drugs targeting them.

## Introduction

GPCRs are the largest transmembrane receptor family in humans, consisting of approximately 800 genes. They are involved in several physiological functions, such as immune response, blood pressure regulation, nerve system, vision system, and olfactory system^1^. Hence, drugs that target GPCRs have been developed to treat multiple human diseases, such as central nervous system disorders, inflammatory diseases, metabolic imbalances, cardiac diseases, and cancer^2,3^.

All GPCRs have conserved seven-pass transmembrane helices (TM 1–7) that are connected by three extracellular loops (ECL 1–3) and three intracellular loops (ICL 1–3). The third intracellular loop (ICL3) is very flexible and interacts with G proteins, which are necessary for intracellular signal transduction. Depending on the conditions within the cells, GPCRs adopt multiple conformational states, with the two major states being the active and inactive states^4^.

During small molecule drug development, the three-dimensional structure of the target protein with the candidate compound is very useful for refining the compound to improve its binding capacity. This method—structure-based drug design (SBDD)—has been widely used since the 1990s^4–6^. Using SBDD, rational drug design is possible, which greatly improves the success rates of drug development. However, structure determination of GPCRs is very difficult due to poor protein expression in native tissues or heterologous expression systems, low protein stability, and the presence of several receptor conformational states^4, 7^. In the last ten years, several technologies on protein expression, purification, crystallisation, and X-ray diffraction data collection have been developed^8–13^. For example, fusion proteins, such as T4 lysozyme^14^ and BRIL^15^, have enabled researchers to purify target GPCR proteins and determine their structures^11^. Our knowledge of GPCR structures has gradually increased, as evidenced by the more than 40 unique GPCR structures that have been determined to date^13^.

To improve the stability of GPCRs for crystallisation, ICLs are often replaced with soluble proteins, such as T4 lysozyme, BRIL, or rubredoxin^11,15^. In cases where the target GPCR cannot be successfully crystallised even after its fusion to soluble proteins, additional approaches are needed to enhance crystallisability, and antibodies that specifically bind to target GPCRs are often used for this purpose. Although this approach is useful to expand the soluble regions of the target for crystal packing^11^, antibodies must be screened for each target GPCR to identify those with high binding affinity, which is very difficult and laborious. Another common approach to increase the thermal stabilities of GPCRs is to mutate the transmembrane helices^3,11^. In this method, the mutation sites are originally specified based on our extensive knowledge of the active and inactive forms of the adenosine A_2a_ receptor. However, screening mutation sites within the target GPCR is still necessary to determine those that stabilise the active or inactive state before the construct can be used for crystallisation experiments. As this approach is also very strenuous and time consuming, there is an urgent need for optimisation methods that are more efficient and can be widely applied to various GPCR targets for crystallography.

Here, we demonstrate that antibodies against fusion proteins can enhance the crystallisation of GPCRs with ligands in active or inactive conformational states. Based on the results presented herein, this approach may be widely applicable for various GPCRs, which would vastly streamline drug development efforts.

## Results

### Sample preparation and crystallisation

The constructs, 5HT_1B_-BRIL/ergotamine (ERG) and AT_2_R-BRIL/[Sar^1^, Ile^8^]-Angiotensin II (s-Ang II), were complexed with SRP2070Fab (the Fab fragment of SRP2070) for crystallography experiments. All crystallisation trials were performed by the lipidic cubic phase (LCP) method^16^. For 5HT_1B_-BRIL/ERG/SRP2070Fab, diffracting crystals grew within 1 week in 400 mM KSCN, 100 mM NaOAc pH 5.5, and 30% PEG 400. For AT_2_R-BRIL/s-Ang II/SRP2070Fab, diffracting crystals grew within 1 week in 50 mM KOAc, 100 mM MES-NaOH pH 6.5, 26–36% PEG 300, and 100 μM s-Ang II.

### Structure determination of 5HT_1B_-BRIL/ERG/SRP2070Fab and AT_2_R-BRIL/s-Ang II/SRP2070Fab

**Figure 1:**
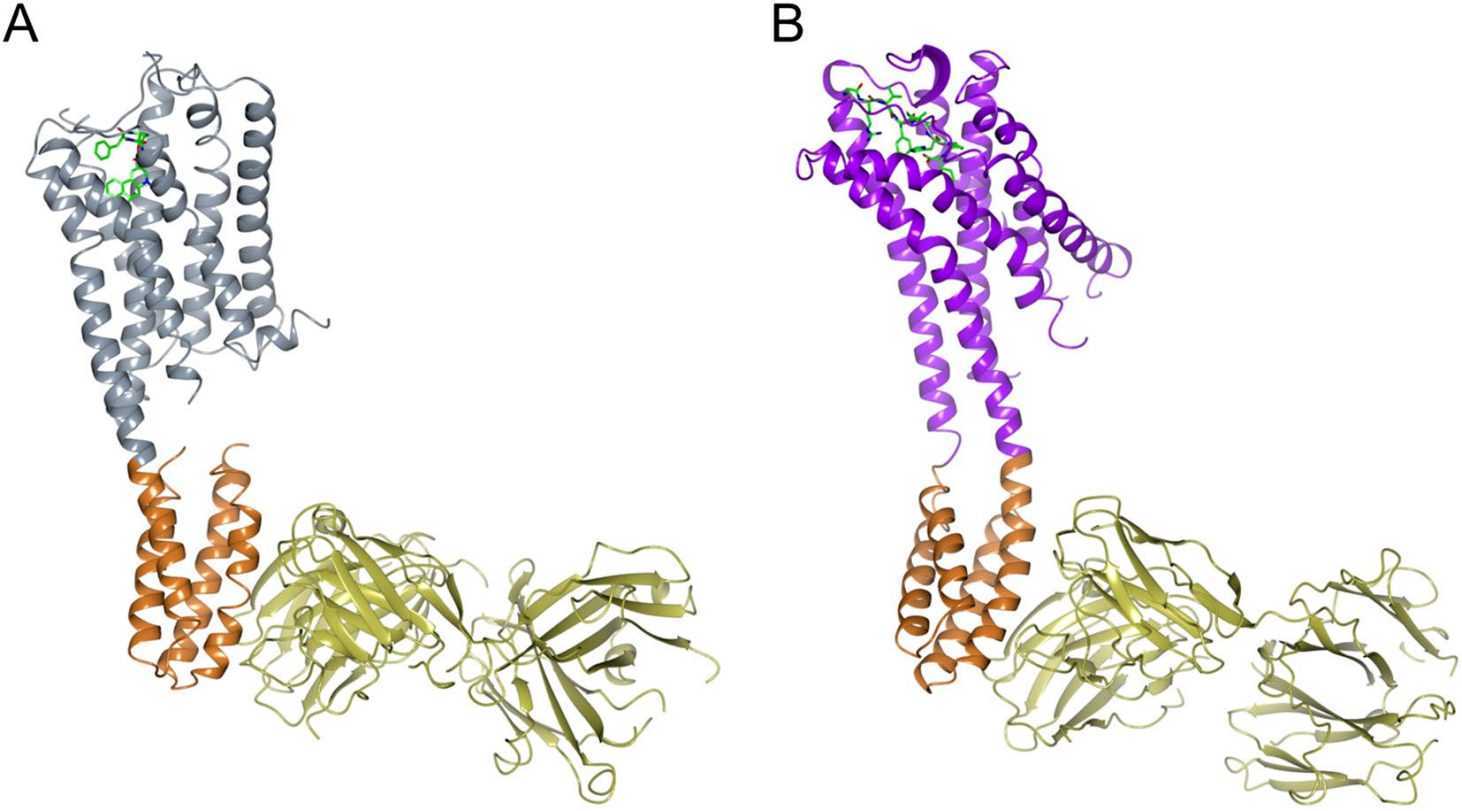
Overall structure of 5HT_1B_-BRIL/ERG/SRP2070Fab and AT_2_R-BRIL/s-Ang II/SRP2070Fab. (**A**) Overall structure of 5HT_1B_-BRIL/ERG/SRP2070Fab. ERG is shown as sticks. Each molecule is coloured as follows: 5HT_1B_ (gray), BRIL (light brown), SRP2070Fab (yellow). (**B**) Overall structure of AT_2_R-BRIL/s-Ang II/SRP2070Fab. s-Ang II is shown as sticks. Each molecule is coloured as follows: AT_2_R (purple), BRIL (light brown), SRP2070Fab (yellow).

**Figure 2:**
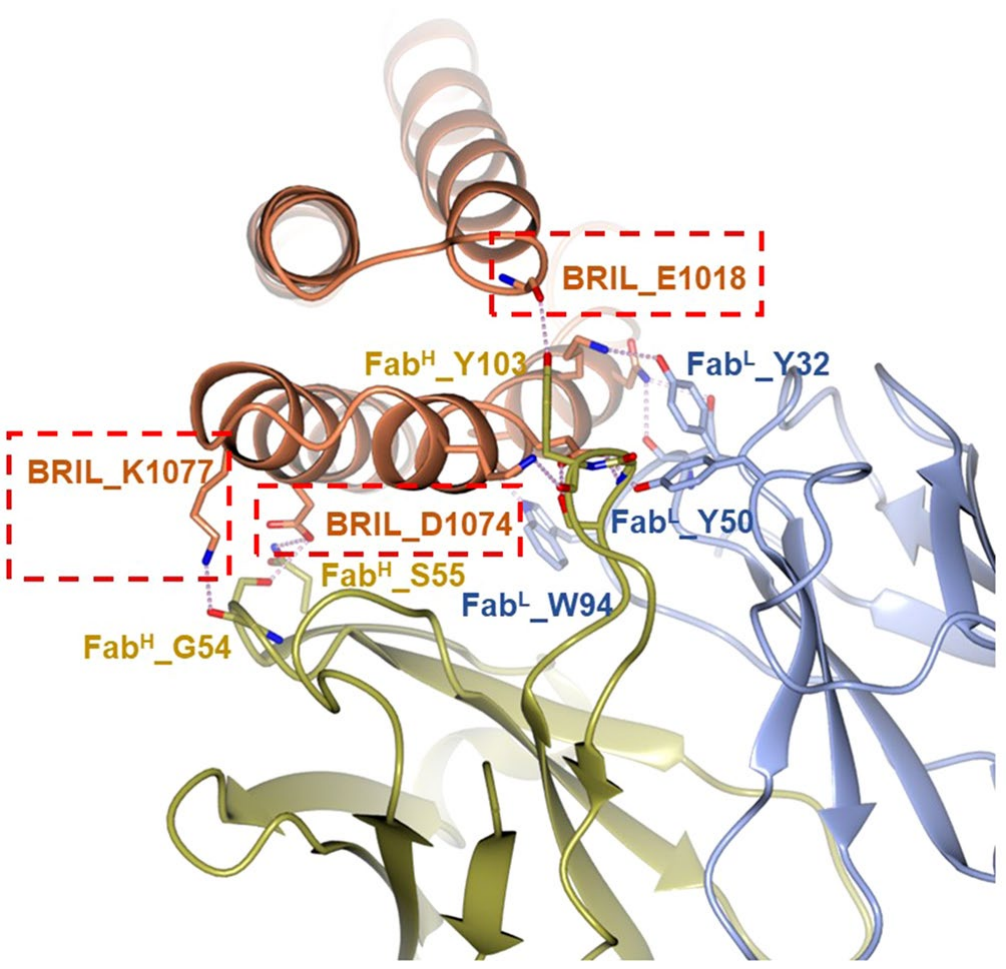
The binding interface between BRIL and SRP2070Fab. Interface-forming residues are coloured as follows: BRIL (brown), SRP2070Fab light chain (dark yellow), and SRP2070Fab heavy chain (light blue). Boxes drawn with red dashed lines indicate residues that are critical for BRIL binding.

The structures of both complexes were solved by molecular replacement and then refined. Following refinement, the *R*_work_/*R*_free_ was 22.2%/28.0% for 5HT_1B_-BRIL/ERG/SRP2070 and 22.6%/27.8% for AT_2_R-BRIL/s-Ang II/SRP2070Fab (Table 1). Figure 1 shows the overall structures of both complexes. SRP2070Fab perpendicularly bound to the BRIL helices. We also observed that helices III and IV of BRIL served as epitopes for SRP2070Fab. In particular, helix III was recognised by both CDR-L3 and CDR-H3, suggesting that this region is a dominant epitope (Fig. 2). These findings indicate that SRP2070 recognises the tertiary structure of BRIL, which may be an important feature of SRP2070Fab as a crystallisation enhancer.

**Table 1:** Data collection and refinement statistics.

	AT_2_R-SRP2070Fab complex (PDB:7C6A)	5HT_1B_-SRP2070Fab complex (PDB:7C61)
**Data collection**		
Space group	*P*2_1_	*C*2
Cell dimensions		
*a*, *b*, *c* (Å)	40.2, 131.0, 113.8	248.8, 65.7, 79.7
*α*, *β*, *γ* (°)	90, 97.1, 90	90, 98.8, 90
Resolution (Å)	42.8–3.4 (3.7–3.4)^a^	48.5–3.0 (3.2–3.00)
*R*_pim_ (%)	14.0 (72.0)	9.0 (61.7)
*I*/*σ* (*I*)	5.1 (1.1)	9.2 (1.6)
*CC*_1/2_	0.99 (0.49)	0.99 (0.62)
Completeness (%)	100.0 (100.0)	100.0 (100.0)
Redundancy	18.8 (14.5)	29.5 (28.2)
**Refinement**		
Resolution (Å)	42.8–3.4 (3.49–3.4)	48.5–3.0 (3.1–3.0)
No. reflections	15,370 (1,120)	24,568 (1,796)
R_work_/R_free_ (%)	22.2/28.0 (37.7/33.2)	21.3/26.2 (32.8/39.7)
No. atoms		
Receptor	2,252	2,105
BRIL	712	719
SRP2070Fab fragment	3,307	3,304
Ligand	69	43
B factors		
Receptor	104.4	158.8
BRIL	110.6	76.9
SRP2070Fab fragment	103.5	68.3
Ligand	104.8	161.5
R.M.S. deviations		
Bond lengths (Å)	0.008	0.011
Bond angles (°)	1.23	1.50
Ramachandran plot		
Favoured (%)	94.5	95.1
Allowed (%)	5.4	4.8
Disallowed (%)	0.1	0.1

Data were collected from 144 and 118 crystals of AT_2_R-SRP2070Fab and 5HT_1B_-SRP2070Fab complexes, respectively.

^a^Values in parentheses are for the highest-resolution shell.

**Figure 3:**
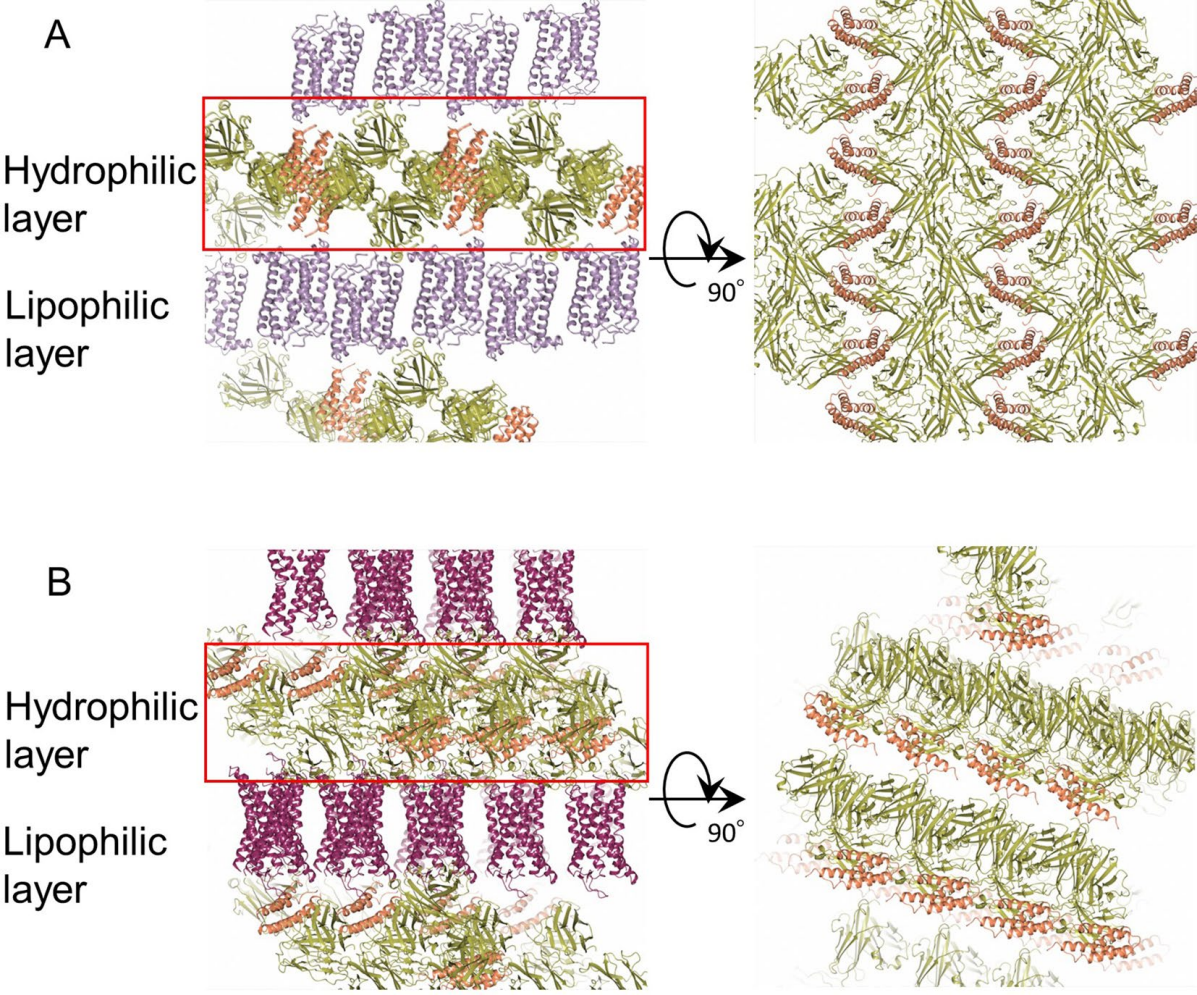
Crystal packing of 5HT_1B_-BRIL/ERG/SRP2070Fab and AT_2_R-BRIL/s-Ang II/SRP2070Fab. (**A**) The crystal packing of 5HT_1B_-BRIL/ERG/SRP2070Fab is shown from two directions. Each molecule is coloured as follows: 5HT_1B_ (light purple), BRIL (light brown), SRP2070Fab (green). (**B**) The crystal packing of AT_2_R-BRIL/s-Ang II/SRP2070Fab is shown from two directions. Each molecule is coloured as follows: AT_2_R (purple), BRIL (light brown), SRP2070Fab (green).

To assess the effect of SRP2070Fab on crystallisation, the crystal packing interactions were investigated. As shown in Fig. 3, crystals of 5HT_1B_-BRIL/ERG/SRP2070Fab and AT_2_R-BRIL/s-Ang II/SRP2070Fab were formed by alternative stacking of the aqueous layers composed of SRP2070Fab and BRIL and the lipophilic layers composed of the receptor region. Crystal packing contacts were observed in the aqueous layers between SRP2070Fab molecules and also within the receptor region in the lipophilic layers. The crystal packing calculations for 5HT_1B_-BRIL/ERG/SRP2070Fab and AT_2_R-BRIL/s-Ang II/SRP2070Fab crystals are shown in Supplementary Table 1S and Supplementary Fig. 4S. In 5HT_1B_-BRIL/ERG/SRP2070Fab crystal, the accessible surface areas (ASA) buried due to crystal packing were 2,206.9 Å^2^ (5HT_1B_), 713.4 Å^2^ (BRIL), and 2,225.2 Å^2^ (SRP2070Fab). The 5HT_1B_-BRIL structure formed a head-to-tail dimer, which is often observed in GPCR crystals similar to the previously reported SRP2070Fab-free 5HT_1B_-BRIL/ERG structures (PDB ID: 4IAR) (Supplementary Fig. 5S). When the crystal packing contribution resulting from the head-to-tail dimerisation was excluded, it was clear that SRP2070Fab was the most dominant crystallisation promoter. These were also observed for AT_2_R-BRIL/s-Ang II/SRP2070Fab. The buried ASAs were 1,329.7 Å^2^ (AT_2_R), 510.1 Å^2^ (BRIL), and 2,293.8 Å^2^ (SRP2070Fab), suggesting that the crystal contacts among SRP2070Fab are the most dominant factor for the crystal formation.

**Figure 4:**
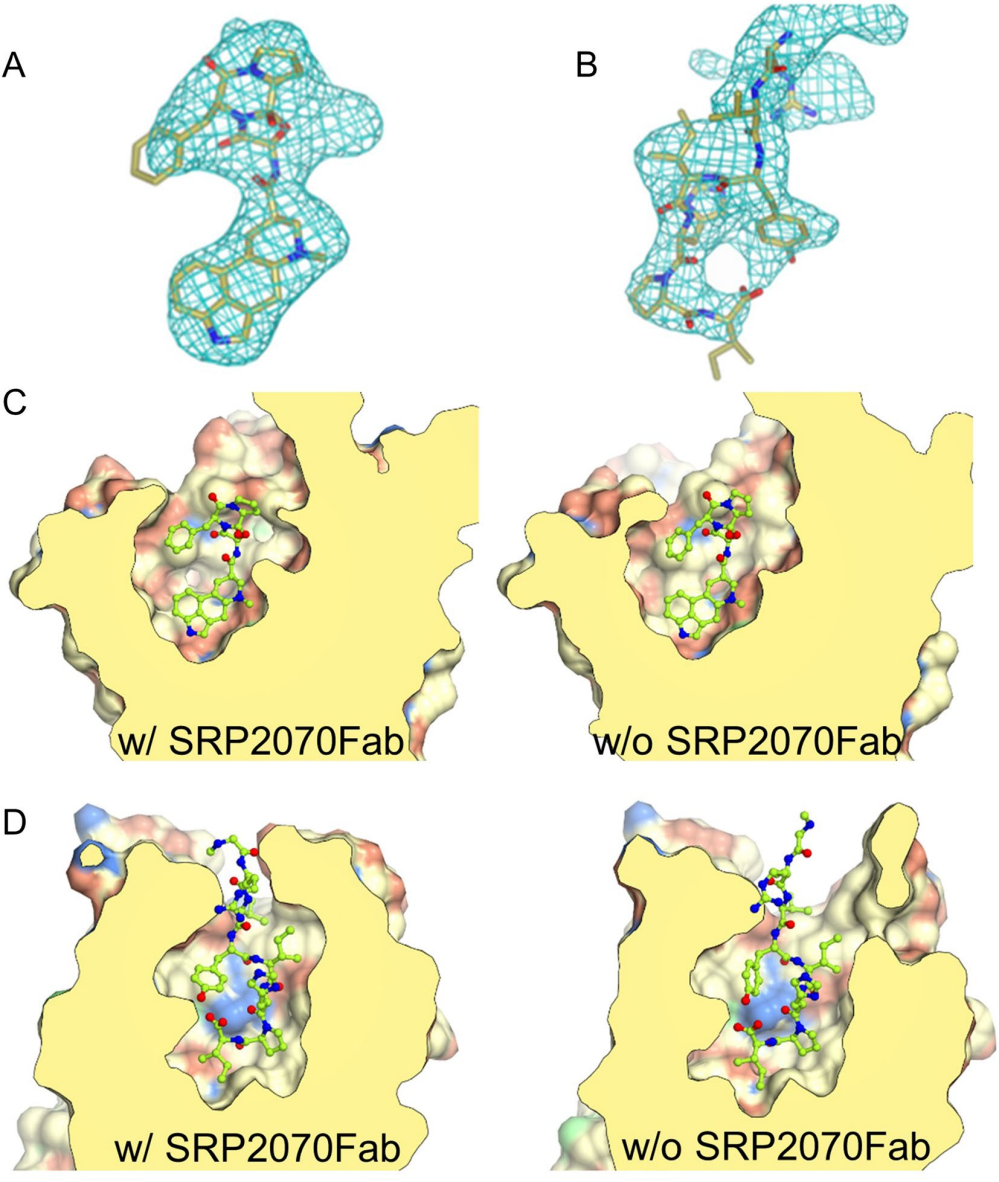
Electron density maps of the ligand and structural comparison of the ligand binding site (**A**) The ligand omitted *mF*o–D*F*c polder electron density (light blue mesh) of ergotamine (ERG) contoured at 3.0 σ. (**B**) The ligand omitted *mF*o–D*F*c polder electron density (light blue mesh) of s-Ang II contoured at 3.0 σ. (**C**) Comparison of ligand binding cavity of 5HT_1B_/ERG/SRP2070Fab (left) and 5HT_1B_/ERG (PDB ID: 4IAR) (right). ERG is shown as sticks. (**D**) Comparison of ligand bindin cavity of AT_2_R/s-Ang II/SRP2070Fab (left) and AT_2_R/s-Ang II (PDB ID: 5XJM) (right). s-Ang II is shown as sticks.

Unambiguous electron densities corresponding to the ligands, ERG for 5HT_1B_ and s-Ang II peptide for AT_2_R, were also observed within the orthosteric binding pockets of the receptors (Fig. 4A,B).

### Structure comparison

To confirm whether the structures of GPCR portion are affected by SRP2070 binding to BRIL, the structures of 5HT_1B_-BRIL/ERG/SRP2070Fab and AT_2_R-BRIL/s-Ang II/SRP2070Fab were compared to the previously reported structures of 5HT_1B_-BRIL/ERG (PDB ID: 4IAR)^17^ and AT_2_R-BRIL/s-Ang II in complex with an anti-AT_2_R antibody (PDB ID: 5XJM)^18^. Superimpositions of these structures showed that SRP2070Fab bound to BRIL had very little influence on the GPCR structures; i.e., the root mean square deviations (RMSDs) within the transmembrane regions of the GPCRs were 0.55 Å for 5HT_1B_-BRIL/ERG and 0.67 Å for AT_2_R-BRIL/s-Ang II between equivalent Cα atoms. Furthermore, no significant differences were found around the ligand binding sites in both AT_2_R-BRIL/s-Ang II and 5HT_1B_-BRIL/ERG as a result of SRP2070Fab binding (Fig. 4C,D). The three activation motifs—called microswitches—PIF^19^, NPxxY^20^, and DRY^20^, were also compared. It was confirmed that the three motifs showed very similar conformations in both comparisons (Supplementary Fig. 1S). Thus, the binding of SRP2070Fab to ICL BRIL fused GPCR has no significant effect on microswitch conformations. And also, it was clearly demonstrated that the activation motifs of AT_2_R-BRIL/s-Ang II in complex with an anti-AT_2_R antibody show an active state in the previous report^18^. Therefore, it is thought that AT_2_R-BRIL/s-Ang II/SRP2070Fab is also in an active form even though BRIL is fused to ICL3. The example of the structure that an agonist binds to the ICL3 BRIL fused GPCR has been already reported. The crystal structure of an active-state human angiotensin II (AngII) type I receptor (AT1R) bound to an AngII analog with partial agonist activity was determined even if BRIL was fused to ICL3^21^. Hence, it is possible that the structure of AT_2_R-BRIL/s-AngII/SRP2070Fab shows an active form. It means that the SRP2070Fab is applicable to active state GPCR structure determination. On the other hand, it is very difficult to state clearly whether the 5HT_1B_-BRIL/ERG/SRP2070Fab and 5HT_1B_-BRIL/ERG show an active or an inactive form. Although the orientation of PIF motif was coincide with that of 5HT_2B_-BRIL structure in complex with ERG (PDB ID: 5TUD)^22^ whose structure was clearly demonstrated as an active form by comparing with the structure of β_2_ adrenergic receptor (β_2_AR)-Gs complex (PDB ID: 3SN6)^22^, the NPxxY and DRY motifs were different from 5HT_2B_-BRIL/ERG (Supplementary Fig. 2SA), which means 5HT_1B_-BRIL/ERG/SRP2070Fab structure does not show an active form. Furthermore, the three motifs of 5HT_1B_-BRIL/ERG/SRP2070Fab structure were not coincide with those of β_2_AR bound to the partial inverse agonist carazolol (PDB ID: 2RH1)^23^ that was known as an inactive form (Supplementary Fig. 2SB), which suggests that 5HT_1B_-BRIL/ERG/SRP2070Fab structure is not a complete inactive form. However, the orientation of TM6 helixes of 5HT_1B_-BRIL/ERG/SRP2070Fab showed more similar to that of carazolol bound β_2_AR than that of 5HT_2B_-BRIL/ERG (Supplementary Fig. 3S).

Based on these comparisons, the 5HT_1B_-BRIL/ERG/SRP2070Fab structure adopts an intermediate active state similar to that of the reported SRP2070Fab-free 5HT_1B_-BRIL/ERG structure. This is especially apparent based on the orientation of this intracellular region of TM6, which adopts a conformation observed in the inactive structure rather than active 5HT_2B_ structure. Outward displacement of intracellular region of TM6 is known to be a structural hallmark of the inactive to active state transition, which results in formation of the cavity that recruits G proteins^13^. Thus, the orientation of fused-BRIL between TM5 and TM6 corresponding to the position of ICL3 is directly affected by the active or inactive state of the GPCR.

## Discussion

In this study, we demonstrate that specific binders, such as antibodies, against soluble fusion partners of GPCRs can facilitate GPCR crystallisation. We discovered a new anti-BRIL antibody (SRP2070Fab), which promoted crystallisation of ICL BRIL-fused GPCRs. Three ICL BRIL-fused GPCR structures were solved in complex with SRP2070Fab. Two of them, 5HT_1B_-BRIL with ERG and AT_2_R-BRIL with s-Ang II, are described in this study; the other, ChemR23-BRIL apo form whose structure has not been reported yet, will be reported in a future manuscript. These results suggest that SRP2070Fab is a versatile tool for promoting the crystallisation and structure determination of various ICL BRIL-fused GPCRs. Importantly, this method of using ICL BRIL-fused proteins in complex with SRP2070Fab could also be used to solve the structures of previously unsolved GPCRs.

Based on our results, SRP2070Fab appears to promote crystallisation by affecting crystal packing. For 5HT_1B_-BRIL/ERG/SRP2070Fab, the overlapping layers of SRP2070Fab formed strong crystal packing by interacting at multiple sites (Fig. 3A). This was also observed in the crystal structure of AT_2_R-BRIL/s-Ang II SRP2070Fab (Fig. 3B). The crystal contacts between SRP2070Fab molecules were the most dominant contacts in both structures, suggesting that the range of the crystallisation conditions for ICL BRIL-fused GPCRs with SRP2070Fab is likely narrow. Thus, we predict that this system will increase the success rate of crystallisation and reduce the sample volume for crystallisation trials. Although we think the SRP2070Fab could be used for N- or C-terminal BRIL fused GPCR, further studies will be needed to demonstrate that.

Co-crystallisation with SRP2070Fab not only improves the success rate of crystallisation, but it is also advantageous for the structure determination. The phasing power of molecular replacement generally depends on the molecular mass of known search models (BRIL/SRP2070Fab in this study) relative to that of the protein of interest. Thus, the phasing power increases as a result of forming a complex with SRP2070Fab (~ 50 kDa) compared to performing phasing with BRIL (~ 12 kDa) alone. For both 5HT_1B_-BRIL/ERG/SRP2070Fab and AT_2_R-BRIL/s-Ang II/SRP2070Fab, phasing by molecular replacement with BRIL/SRP2070Fab resulted in electron densities that were interpretable even in the GPCR portion.

**Figure 5:**
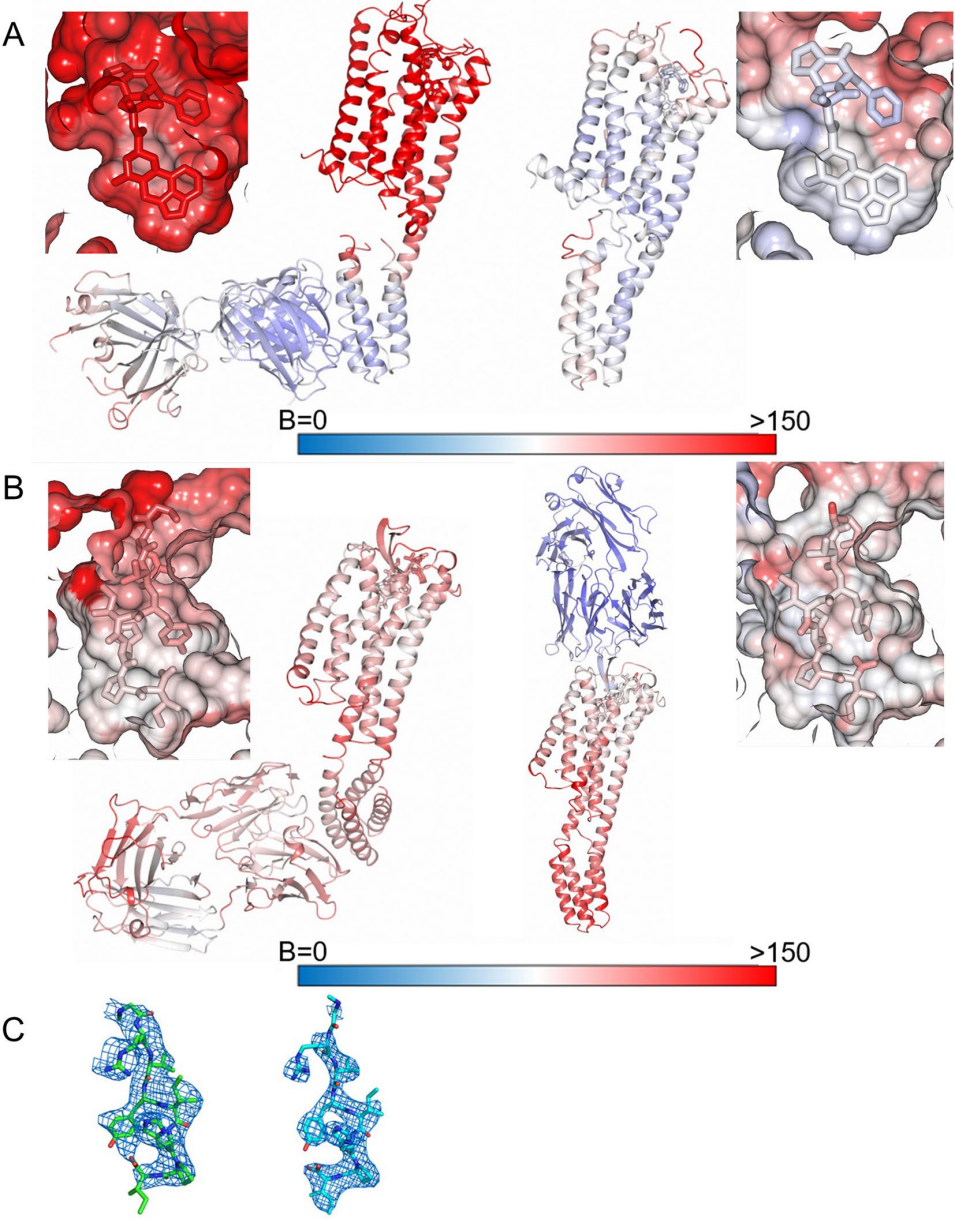
B-factor analysis and comparison of the ligand electron density maps. (**A**) B-factor analysis for 5HT_1B_/ERG/SRP2070Fab (left) and 5HT_1B_/ERG (PDB ID: 4IAR) (right). Both the overall structure and the area around ligand are shown. (**B**) B-factor analysis for AT_2_R-BRIL/s-Ang II/SRP2070Fab (left) and AT_2_R/s-Ang II (PDB ID: 5XJM) (right). Both the overall structure and the area around ligand are shown. (**C**) The ligand electron density map of AT_2_R-BRIL/s-Ang II/SRP2070Fab (left) and AT_2_R/s-AngII (PDB ID: 5XJM) (right). The omitted m*Fo*–D*Fc* electron density map contoured at 3.0 σ (Polder map).

To use the SRP2070Fab for structure-based drug design (SBDD), it is very important to confirm that the SRP2070Fab does not adversely affect the electron density of the ligand. As demonstrated in a previous study, antibodies recognising ICLs are not suitable for SBDD, because the electron density of the ligand tends to be unclear^24^. BRIL is often inserted in place of ICL3 to stabilise GPCRs^11^, and thus SRP2070Fab also binds to the intracellular portion. To determine whether SRP2070Fab is available for SBDD, the B-factor and the ligand density map of structures in this study were compared with the previously reported structures, 5HT_1B_-BRIL/ERG (4IAR) and AT_2_R-BRIL/s-Ang II in complex with anti AT_2_R antibody (5XJM) (Fig. 4). For 5HT_1B_-BRIL/ERG/SRP2070Fab, although the B-factor was higher than that observed in 5HT_1B_-BRIL/ERG (4IAR) (Fig. 5A), the ligand electron density in the OMIT map^25,26^ was clearly recognised and was similar to that of 5HT_1B_-BRIL/ERG (4IAR) (Fig. 4A). Regarding AT_2_R-BRIL/s-Ang II/SRP2070Fab, the B-factors showed a little bit higher in the GPCR part (Fig. 5B), and the ligand electron density in the OMIT map was as clear as that of AT_2_R-BRIL/s-Ang II in complex with anti AT_2_R antibody (5XJM) (Fig. 4B). Thus, the ligand electron density maps between the structures with SRP2070Fab (intra-cellular recognition) and the antibody recognising extra-cellular domain (5XJM) were in good agreement (Fig. 5C). The B-factor of the GPCR parts of our structures are higher than the previously reported structures (Figs. 5A,B). It is because the interaction between SRP2070Fabs has the important role in the crystal packing and the interaction between GPCRs is relatively weak as shown in Fig. 3. However, we think this property is necessary to the versatility of SRP2070Fab. In short, although the SRP2070Fab do not give effects on GPCR structures, it can improve the crystal packing.

Based on these results, SRP2070Fab does not appear to affect ligand binding, indicating that SRP2070Fab can be used for SBDD. Considering the versatility of SRP2070Fab, we anticipate that this antibody will be beneficial for performing SBDD applications. Because SRP2070Fab recognises only BRIL, it is thought that it has no effect on the GPCR; this would suggest that BRIL may absorb any structure fluctuations caused by binding of SRP2070Fab. While 5HT_1B_/ERG and BRIL were linearly aligned through TM5 and TM6 and SRP2070Fab was vertically bound to BRIL, AT_2_R/s-Ang II and BRIL were unaligned by ~ 30°, and SRP2070Fab was vertically bound to BRIL (Fig. 1). This result suggests that there is some structural flexibility between GPCR and BRIL when SRP2070Fab functions as a crystallisation enhancer. Because of this property and the fact that SRP2070Fab is applicable to structure solution of 5HT_1B_ in intermediate active state, which is rather similar to inactive state structure, as well as active AT_2_R, it is reasonable to hypothesize that SRP2070Fab can be used to study both active and inactive states. This is another important feature of SRP2070 as is applicable to both of agonist and antagonist drug developments.

In recent years, the resolution of cryo-electron microscopy has improved, and several structures of GPCRs have been determined by this technique^27^. We anticipate that SRP2070Fab could also be used for cryo-EM analyses. By attaching SRP2070Fab to BRIL-fused GPCRs, the molecular weight can be increased and the SRP2070Fab shape may be useful as a marker for 2D image classification and averaging.

To our knowledge, the use of an antibody like SRP2070Fab to promote crystallisation has not been previously reported. This method may facilitate the structural study of many types of GPCRs. Considering the results described herein, SRP2070Fab is a promising structural biology tool for studying GPCRs and could be useful for developing drugs that target GPCRs.

## Methods

### Ethics statement

Mice experiments in this research were conducted in accordance with the Fundamental Guidelines for Proper Conduct of Animal Experiment and Related Activities in Academic Research Institutions under the jurisdiction of the Ministry of Education, Culture, Sports, Science and Technology of Japan. The protocols conducted in this study were reviewed and approved by Kyoto University institutional review board.

### Complete amino acid sequences and constructs for crystallisation

The sequence of 5HT_1B_ residues 33-390 **(**UniProt accession: P28222) with BRIL insertion (underlined)^15^ is provided in Table 2. The additional N-terminal residue retained after cleavage with tobacco etch virus (TEV) is italicised.

**Table 2:** The amino acids sequences.

5HT_1B_-BRIL	*G*CSAKDYIYQDSISLPWKVLLVMLLALITLATTLSNAFVIATVYRTRKLHTPANYLIASLAVTDLLVSILVMPISTMYTVTGRWTLGQVVCDFWLSSDITCCTASIWHLCVIALDRYWAITDAVEYSAKRTPKRAAVMIALVWVFSISISLPPFFWRQAKAEEEVSECVVNTDHILYTVYSTVGAFYFPTLLLIALYGRIYVEARSRIADLEDNWETLNDNLKVIEKADNAAQVKDALTKMRAAALDAQKATPPKLEDKSPDSPEMKDFRHGFDILVGQIDDALKLANEGKVKEAQAAAEQLKTTRNAYIQKYLAARERKATKTLGIILGAFIVCWLPFFIISLVMPICKDACWFHLAIFDFFTWLGYLNSLINPIIYTMSNEDFKQAFHKLIRFKCTS
AT_2_R-BRIL	*G*CSQKPSDKHLDAIPILYYIIFVIGFLVNIVVVTLFCCQKGPKKVSSIYIFNLAVADLLVLATLPLWATYYSYRYDWLFGPVMCKVFGSFLTLNMFASIWFITCMSVDRYQSVIYPFLSQRRNPWQASYIVPLVWCMACLSSLPTFYFRDVRTIEYLGVNACIMAFPPEKYAQWSAGIALMKNILGFIIPLIFIATCYFGIRKHLLKTNADLEDNWETLNDNLKVIEKADNAAQVKDALTKMRAAALDAQKATPPKLEDKSPDSPEMKDFRHGFDILVGQIDDALKLANEGKVKEAQAAAEQLKTTRNAYIQKYLKNRITRDQVLKMAAAVVLAFIICWLPFHVLTFLDALAWMGVINSCEVIAVIDLALPFAILLGFTNSCVNPFLYCFVGNRFQQKLRSVFRVPITWLQGKRES*ENLYFQ*
The variable region of the SRP2070 light chain	DIVLTQSPATLSVTPGDRVSLSCRASQSVSNYLHWYQQKSHESPRLLIKYASQSISGIPSRFSGSGSGTDFTLSINSVETEDFGMYFCQQSNSWPLTFGAGTKLELR
The variable region of the SRP2070 heavy chain	QIQLQQSGPELVKPGASVKISCKASGYTFTDFYINWMKQRPGQGLEWIGWIFPGSGNTHYNEKFKGKATLIVDTSSSTAFMQLNSLTSEDSAVYFCTRPVSYYYDFDYWGQGTTLTVSS

The sequence of AT_2_R residues 35-346 (UniProt accession: P50052) with the BRIL insertion^15^ (underlined) is provided in Table 2. The mutations, L93V and F133W, were added to improve stabilisation. The additional N- and C-terminal residues retained after cleavage with tobacco etch virus (TEV) cleavage are italicised.

The subclass of the SRP2070 was a mouse IgG2a. The sequence of the variable region of the light chain is provided in Table 2; CDR-L1, CDR-L2, and CDR-L3 are underlined.

The sequence of the variable region of the heavy chain is provided in Table 2; CDR-H1, CDR-H2, and CDR-H3 are underlined.

Codon-optimised cDNA encoding *H. sapiens* AT_2_R-BRIL and 5HT_1B_-BRIL was cloned into pFastBac1 (Thermo Fisher Scientific, Waltham, MA, USA). For AT_2_R-BRIL, the sequences encoding haemagglutinin (HA) and FLAG tags followed by a TEV cleavage site were added to the N-terminus, and a TEV cleavage site followed by a His_8_ tag were added to the C-terminus. For 5HT_1B_-BRIL, haemagglutinin (HA), FLAG, and His_8_ tags followed by a TEV cleavage site were added to the N-terminus.

### Culture and membrane preparation

AT_2_R-BRIL was expressed in *Spodoptera frugiperda* (*Sf*9) cells using the baculovirus-based expression system (cell density = 2.0–4.0 × 10^6^ cells/mL; MOI = 0.5; expression time = 60 h). The culture was harvested by centrifugation (7,000 × *g*, 10 min, 4 °C), and cell pellets were flash frozen at − 80 °C. Frozen cells were thawed and suspended in the hypotonic buffer (10 mM HEPES pH 7.5, 20 mM KCl, 10 mM MgCl_2_, 1 × protein inhibitor cocktail tablet). The suspension was centrifuged, and the pellet was resuspended in high osmotic buffer [10 mM HEPES pH 7.5, 10 mM MgCl_2_, 20 mM KCl, 1 M NaCl, 1 × protease inhibitor cocktail (Sigma Aldrich, Saint Louis, MO, USA)]. The cells were lysed using a dounce homogeniser, and the lysate was centrifuged (100,000 × *g*, 30 min, 4 °C). The pellet was resuspended in high osmotic buffer containing 40% glycerol and stored at − 80 °C. Plasma membranes expressing 5HT_1B_-BRIL were prepared using the same method as for AT_2_R-BRIL.

### Production of monoclonal antibody fragment (SRP2070Fab)

Purified GPCR-BRIL proteins were reconstituted into liposomes consisting of 20:1 (w/w) egg phosphatidylcholine (Sigma-Aldrich)/monophosphoryl lipid A (Sigma-Aldrich). MRL/lpr mice were immunised with liposomal GPCR-BRIL. After immunisation, the mouse spleens were removed, and spleen cells were fused with myeloma cells. Hybridoma cells were screened by liposome-ELISA, denatured dot blot analysis, and fluorescence size exclusion chromatography (FSEC). To screen antibodies that specifically bind to the antigen with correct structure, but not the denatured one, liposome-ELISA positive and denatured dot blot clones were pooled, and then we confirmed the binding capacity in aqueous solution by FSEC. Hybridoma cells producing SRP2070 were intraperitoneally administered to mice, and ascites were collected. SRP2070 was purified by ammonium sulphate precipitation and protein G column chromatography. The subclass of SRP2070 was mouse IgG2a. After cleaving SRP2070 with papain, SRP2070Fab was purified by protein A column chromatography to remove the Fc portion and further purified by size exclusion chromatography (Superdex200 10/300 GL, GE Healthcare) by the changing buffer into PBS.

### Purification of 5HT_1B_-BRIL/ERG/SRP2070Fab

The construct for structural analysis was expressed in accordance with a previous study^17^. The amino acids from Leu240 to Met305 were replaced with BRIL. In addition, the glycosylation site was removed by deleting 32 amino acid residues at the N-terminus. The HA signal sequence, FLAG tag, His_10_-tag, and TEV protease recognition site were added. Furthermore, the L138W mutation was introduced to increase thermal stability. The DNA coding sequence was inserted into pFastBac1 using BamHI and HindIII.

Purified plasma membranes were thawed on ice and resuspended in solubilisation buffer (50 mM HEPES pH 7.5, 500 mM NaCl, 10% glycerol, 20 mM imidazole, 1% n-dodecyl-β-d-maltoside (DDM), 0.2% cholesteryl hemisuccinate (CHS), 1 × protein inhibitor cocktail) supplemented with 100 mg/mL iodoacetamide (Wako Pure Chemical Industries, Ltd.) and 50 μM ERG (Sigma Aldrich, Saint Louis, MO, USA), and then solubilised by rotating gently for 3 h at 4 °C. The supernatant was isolated by centrifugation at 235,000 × *g* for 45 min and incubated with TALON Superflow Metal Affinity Resin (Clontech; 1 mL of resin per 500 mL of original culture volume was used) for 2 h at 4 °C. After incubation, the resin was washed with five column volumes of wash buffer (50 mM HEPES pH 7.5, 500 mM NaCl, 10% glycerol, 0.1% DDM, 0.02% CHS, 20 mM imidazole, 50 μM ERG, and 10 mM ATP), followed by five column volumes of wash buffer containing 800 mM NaCl. 5HT_1B_-BRIL protein was eluted with three column volumes of elution buffer (50 mM HEPES pH 7.5, 500 mM NaCl, 10% glycerol, 0.1% DDM, 0.02% CHS, 500 mM imidazole, and 50 μM ERG), concentrated, and then buffer exchanged into wash buffer without imidazole. The sample was treated with His-tagged TEV protease (expressed in-house) at 4 °C overnight to remove the N-terminal tags. The His-tagged TEV protease and cleaved N-terminal fragment were removed using Ni–NTA fast flow resin (GE Healthcare). 5HT_1B_-BRIL/ERG complex was collected as the Ni–NTA column flow-through fraction and concentrated to ~ 30 mg/mL using a 100-kDa MWCO Amicon concentrator.

The 5HT_1B_-BRIL/ERG/SRP2070Fab complex was prepared by mixing 5HT_1B_-BRIL/ERG and SRP2070Fab at a molar ration of 1:1.5 for 1 h on ice. The complex was purified by SEC (Superdex 200 increase 200, GE healthcare). Peak fractions containing 5HT_1B_-BRIL/ERG/SRP2070Fab were collected and concentrated to 30 mg/mL using a 100-kDa MWCO Amicon concentrator and then used for crystallisation experiments.

### Crystallisation and data collection of 5HT_1B_-BRIL/ERG/SRP2070Fab complex

5HT_1B_-BRIL/ERG/SRP2070Fab were reconstituted into LCP by mixing with host lipids (monoolein:cholesterol = 9:1) at a protein:lipid ratio (v:w) of 2:3 using a mixer consisting of two 100 μL gas tight syringes (Hamilton Company). Crystal trays (96 well glass sandwich plates), containing 30 nL LCP sample covered by 800 nL mother liquor per well, were set up using a Gryphon LCP crystallisation robot (Art Robbins Instruments) and then incubated at 20 °C. Droplets were imaged chronologically by RockImager 1,000 (FORMULATRIX, Bedford, MA). Crystals were obtained from precipitant conditions containing 0.4 M KSCN, 0.1 M NaOAc pH 5.5, and 30% PEG 400. Plate-shaped crystals grew to a maximum size of 30 × 20 × 5 μm^3^ in 3‒4 days, were harvested using MiTeGen MicroMounts (MiTeGen), and flash frozen in liquid nitrogen. X-ray data were collected at the BL32XU beamline at SPring-8 (Japan Synchrotron Radiation Research Institute). Most crystals diffracted to ~ 3.0 Å resolution (1.0 s exposure, 1.0° oscillation, 10 μm beam attenuated by a 500‒1,200 μm aluminium sheet). To reduce radiation damage, crystals were exchanged after every 5°–10° of data collection. A complete data set at 3.0 Å resolution was obtained by merging the individual data sets collected from 144 crystals using KAMO^28^.

The initial structures were solved by molecular replacement as follows: as MR search models, the BRIL/SRP2070 complex (solved in-house) structure was separated into BRIL/Fv region and Fc region, and the receptor region of 5HT_1B_ was clipped from 5HT_1B_-BRIL structure (PDB ID: 4IAR). The MR search was performed with *MOLREP*^29^ using three search models. Rigid-body and restrained refinements were performed with *REFMAC5*^30^. The ligand was placed in the electron density and the model was corrected with *COOT*^31^. The polder ligand omit map 26 was calculated with *PHENIX*^32^.

### Purification of AT_2_R-BRIL/s-Ang II/SRP2070Fab

Residues 1–34 and 347–363 of human AT_2_R were deleted, and BRIL was inserted between N242 and K246 of AT_2_R. The HA signal sequence, FLAG tag, and TEV protease recognition site were added to the N-terminus, and TEV protease recognition site and His_8_ tag were added to the C-terminus of AT_2_R. The membrane fraction purified from the *Sf*9 cell pellet was solubilised and incubated (1 h, 4 °C) with solubilisation buffer (50 mM HEPES pH 7.5, 800 mM NaCl, 10% glycerol, 1% DDM, 0.2% CHS, and 15 mM imidazole) supplemented with 100 mg/mL iodoacetamide (Wako Pure Chemical Industries, Ltd.) and 200 μM s-Ang II (Peptide Institute Inc.). After centrifugation (100,000 × *g* for 30 min, 4 °C), the supernatant was incubated with TALON Superflow Metal Affinity Resin (Clontech, 1–2 mL of resin per 500 mL of original culture volume was used) overnight at 4 °C with gentle agitation. After incubation, the resin was washed with twenty column volumes of wash buffer (50 mM HEPES pH 7.5, 200 mM NaCl, 10% glycerol, 0.1% DDM, 0.02% CHS, 15 mM imidazole, and 200 μM s-Ang II), then eluted by five column volumes of elution buffer (50 mM HEPES pH 7.5, 200 mM NaCl, 10% glycerol, 0.03% DDM, 0.006% CHS, 250 mM imidazole, and 200 μM s-Ang II). The eluted protein sample was concentrated using a 100-kDa MWCO Amicon concentrator, and the buffer was exchanged into wash buffer without imidazole.

The AT_2_R-BRIL/s-Ang II/SRP2070Fab complex was prepared by mixing AT_2_R-BRIL/s-Ang II and SRP2070Fab at a molar ratio of 1:1.5 for 1 h on ice. His-tagged TEV protease was added to the sample and incubated 4 °C overnight to remove the N-terminal FLAG tag and C-terminal His_8_-tag. The His_8_-tagged TEV protease and cleaved C-terminal fragment were removed by incubating with 2 mL of Ni–NTA fast flow resin (GE Healthcare) at 4 °C for 1 h. AT_2_R-BRIL/s-Ang II/SRP2070Fab complex was collected as the Ni–NTA column flow-through fraction and concentrated using a 100-kDa MWCO Amicon concentrator. The final complex sample was purified by SEC (Superdex 200 10/300, GE Healthcare). Peak fractions containing AT_2_R-BRIL/s-Ang II/SRP2070Fab were collected and concentrated to ~ 30 mg/mL using a 100-kDa MWCO Amicon concentrator.

### Crystallisation and data collection of AT_2_R-BRIL/s-Ang II/SRP2070Fab complex

AT_2_R-BRIL/s-Ang II/SRP2070Fab and host lipids (monoolein:cholesterol = 9:1) were mixed at a protein:lipid ratio (v:w) of 2:3 using a mixer consisting of two 100 μL gas tight syringes (Hamilton Company). Crystal trays (96-well glass sandwich plates), containing 30 nL LCP sample overlaid by 800 nL mother liquor per well, were set up using a NT-8 crystallisation robot (FORMULATRIX), incubated at 20 °C, and imaged chronologically with a RockImager 1,000.

The crystals were obtained from crystallisation conditions containing 50 mM KOAc, 0.1 mM MES pH 6.5, 26–36% PEG 300, 100 μM s-Ang II. Rod-shaped crystals grew to a maximum size of 30 × 5 × 5 μm^3^ within a week, were harvested using MiTeGen MicroMounts (MiTeGen), and flash frozen in liquid nitrogen. X-ray data were collected at the BL32XU beamline at SPring-8 (Japan Synchrotron Radiation Research Institute). Most crystals diffracted to ~ 3.5 Å resolution (1.0 s exposure, 1.0° oscillation, 10 μm beam attenuated by a 500–1,200 μm aluminium sheet). To reduce radiation damage, crystals were exchanged after every 5°–10° of data collection. Finally, a complete data set at 3.4 Å resolution was obtained by merging the individual data sets collected from 118 crystals using the *KAMO* system^28^.

The initial structure was determined by molecular replacement with *PHASER* using BRIL/SRP2070Fv, SRP2070Fv, and the receptor region of AT_1_R-BRIL (PDB ID: 4ZUD) as search models. Rigid-body and restrained refinements were performed with *REFMAC5*^30^. The ligand was placed in the electron density and the model was corrected with *COOT*^31^. The polder ligand omit map 26 was calculated with *PHENIX*^32^.

The detailed crystal packing calculations for 5HT_1B_-BRIL/ERG/SRP2070Fab and AT_2_R-BRIL/s-Ang II/SRP2070Fab crystal structures were calculated with PISA^33^ and AREAIMOL in CCP4^34,35^ and shown in Supplementary Table 1S and Supplementary Fig. 4S. Structural figures were prepared using the CCP4mg molecular-graphics software^36^ except for Fig. 4C,D. Figure 4C,D were prepared using CueMol (https://www.cuemol.org/).

## Supplementary information

Supplementary Information 1 (PDF 2608 kb)

## Data Availability

Atomic coordinates and structure factors are deposited in the Protein Data Bank under accession codes 7C61 for the 5HT_1B_-BRIL/ERG/SRP2070Fab and 7C6A for the AT_2_R-BRIL/s-Ang II/SRP2070Fab. All of the data and information about constructs used in this study are available upon reasonable request from the corresponding author.
