# Supplementary material for: The discovery of a new antibody for BRIL-fused GPCR structure determination
Source: Sci Rep. 2020 Jul 15;10:11669. doi: 10.1038/s41598-020-68355-x (PMC7363855; doi:10.1038/s41598-020-68355-x)
Supplement: Supplementary file 1 — Supplementary Information 1 (PDF 2608 kb) [file 41598_2020_68355_MOESM1_ESM.pdf]

Supplementary Information

Article title: The discovery of a new antibody for BRIL-fused GPCR structure determination

Authors: Hikaru Miyagi[1, †], Hidetsugu Asada[2, †], Michihiko Suzuki[3], Yuichi Takahashi[3], Mai Yasunaga[3], Chiyo Suno[2], So Iwata[2,4] and Jun-ichi Saito[3*]

Affiliations: [1]R&D Division, Kyowa Kirin Co., Ltd., Tokyo, Japan.
[2]Department of Cell Biology, Graduate School of Medicine, Kyoto University, Kyoto, Japan.
[3]R&D Division, Kyowa Kirin Co., Ltd., Shizuoka, Japan.
[4]RIKEN, SPring-8 Center, Hyogo, Japan.

[†]These authors contributed equally to this work.

*Corresponding author: Jun-ichi Saito
Email: jun.saito.su@kyowakirin.com
Phone: 81-55-989-2004; Fax:81-55-986-7430;

Supplementary Table 1S: The crystal packing calculations

	AT_2R-BRIL/s-ArgII/SRP2070Fab			$5HT_{1B}$-BRIL/s-ArgII/SRP2070Fab		
	AT_2R	BRIL	SRP2070 Fab	$5HT_{1B}$	BRIL	SRP2070 Fab
total SA ($Å^2$)	14983.4	5618.1	19757.9	14822.5	5745.0	19609.3
buried SA by crystal contact ($Å^2$)	1329.7	510.1	2293.8	2206.9	713.4	2225.2

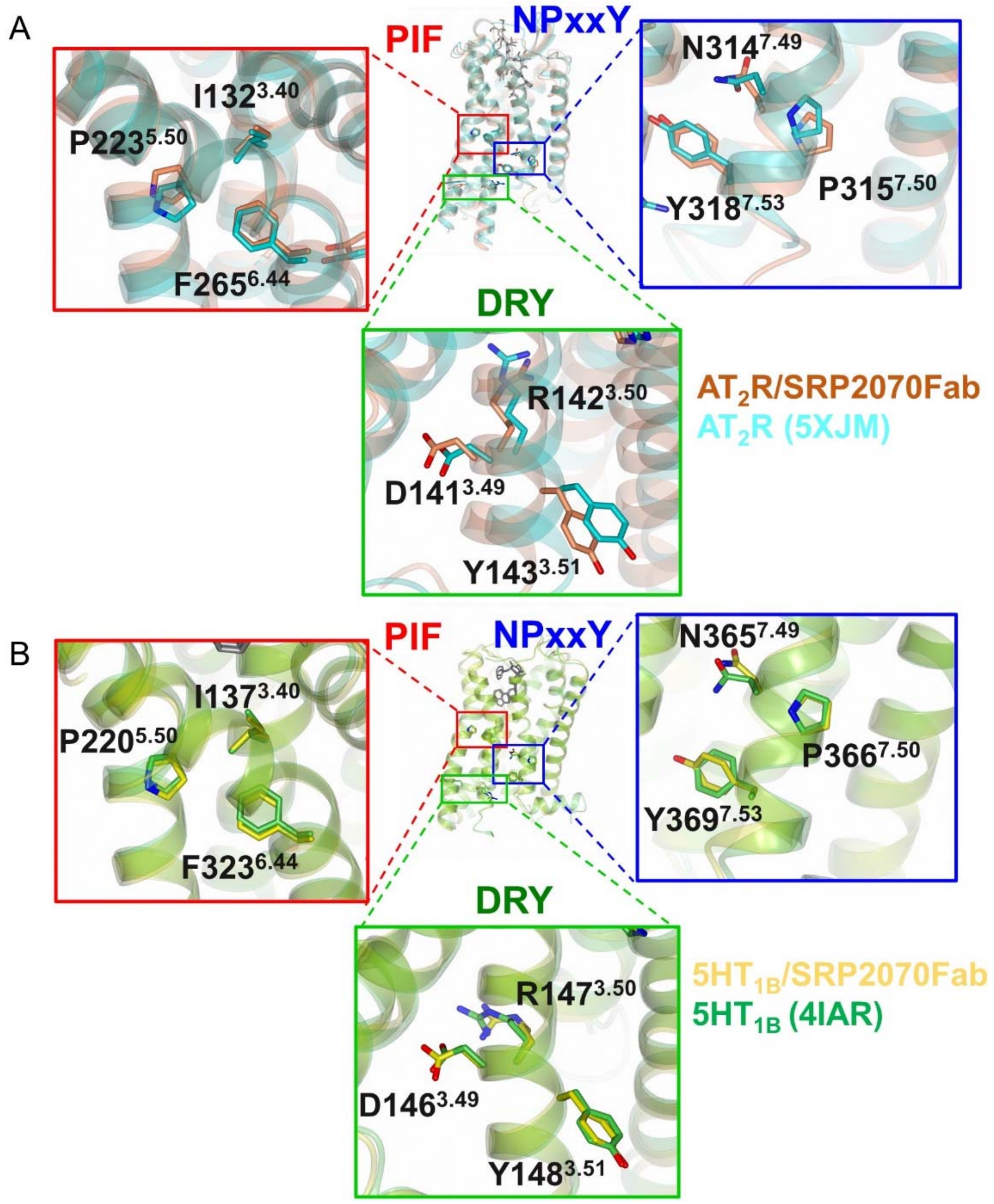

Supplementary Figure 1S: Structural comparison of the activation motifs

(A) Superpositions of AT_2R /s-Ang II /SRP2070Fab (light brown; this study), and AT_2R/s-Ang II with antibody (light blue; PDB ID: 5XJM). The activation motifs PIF, NPxxY, and DRY are shown as red, blue, and green boxes, respectively. (B) Superpositions of $5HT_{1B}$/ERG/SRP2070Fab (yellow; this study), and $5HT_{1B}$/ERG (green; PDB ID: 4IAR). The activation motifs PIF, NPxxY, and DRY are shown as red, blue, and green boxes, respectively.

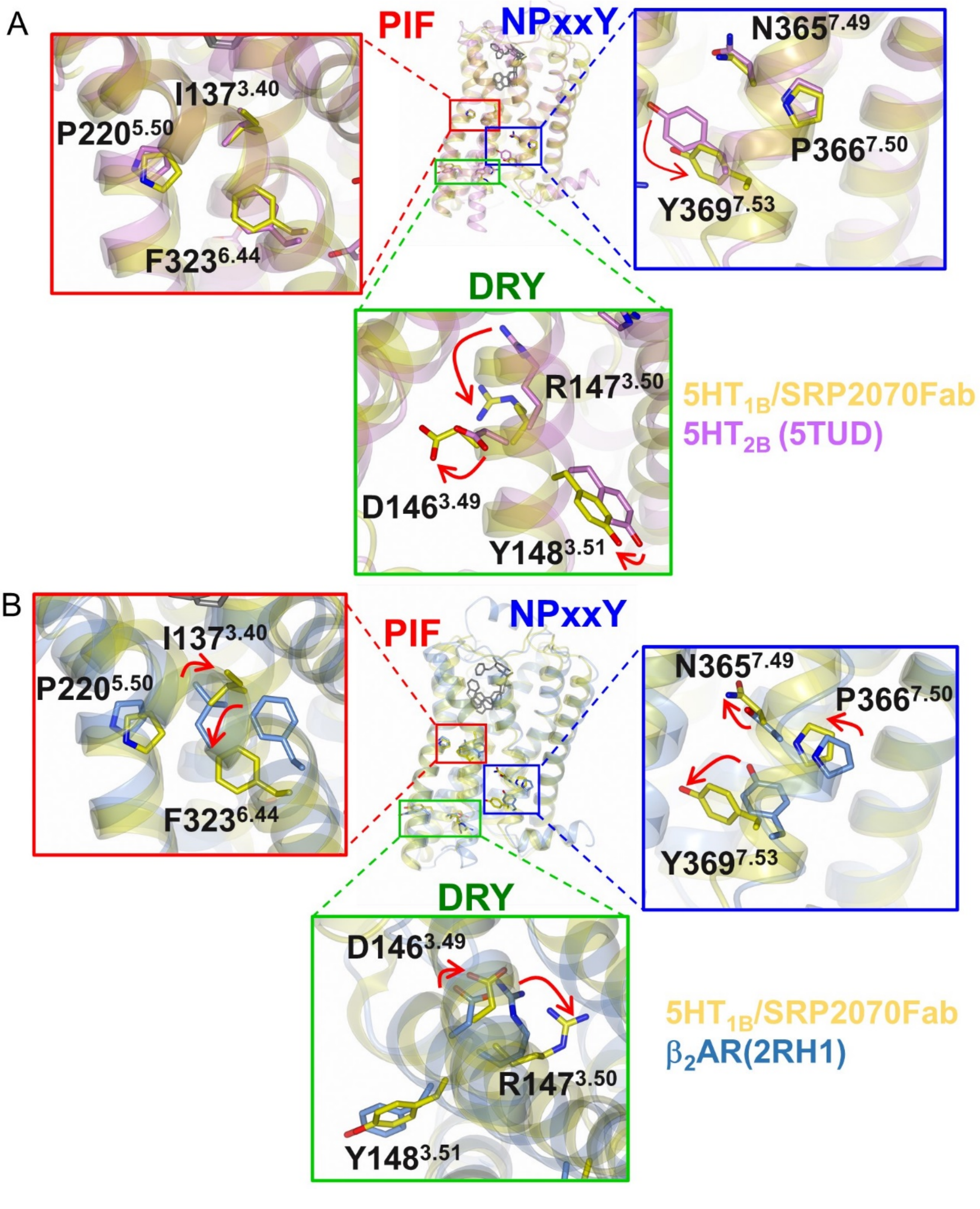

Supplementary Figure 2S: Structural comparison of the activation motifs

(A) Superpositions of $5HT_{1B}$/ERG/SRP2070Fab (yellow; this study), and $5HT_{2B}$/ERG (magenta; PDB ID: 5TUD). The activation motifs PIF, NPxxY, and DRY are shown as red, blue, and green boxes, respectively. (B) Superpositions of $5HT_{1B}$/ERG/SRP2070Fab (yellow; this study), and β_2AR (light blue; PDB ID: 2RH1). The activation motifs PIF, NPxxY, and DRY are shown as red, blue, and green boxes, respectively.

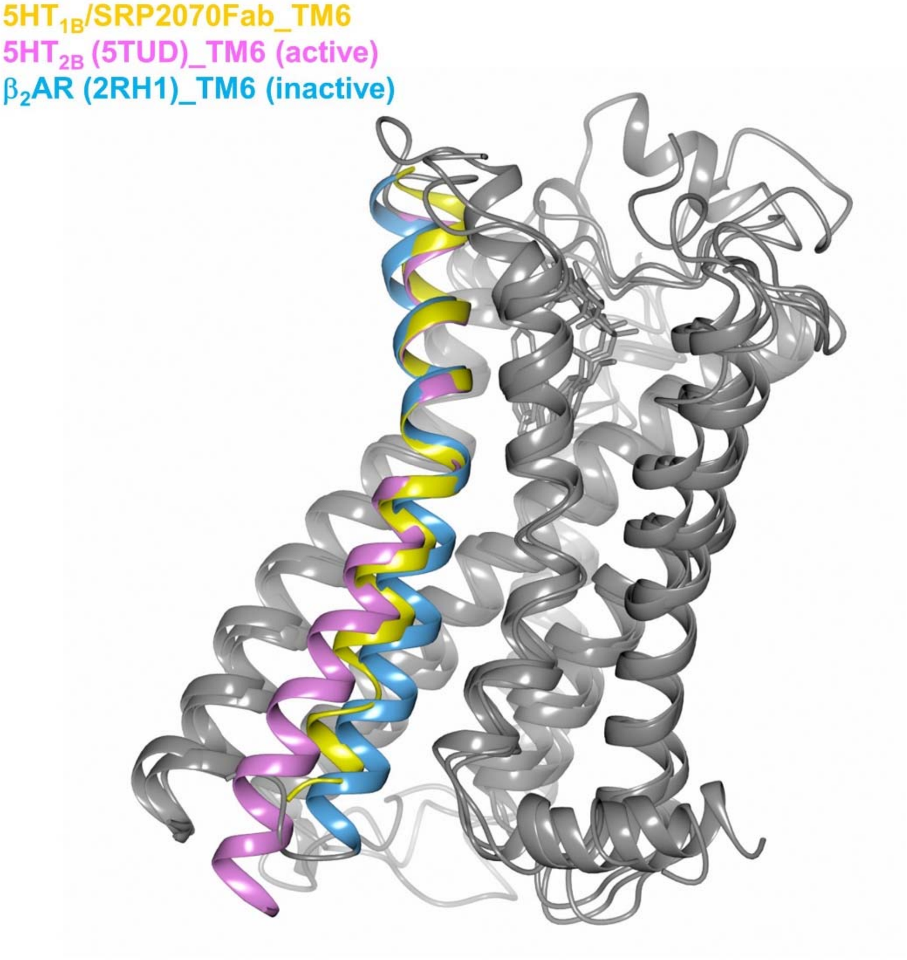

Supplementary Figure 3S: Structural comparison of TM6 helixes

Superpositions of 5HT$_{1B}$/ERG/SRP2070Fab, 5HT$_{2B}$/ERG (PDB ID: 5TUD), and β_2AR (PDB ID: 2RH1). The TM6 helixes are colored, 5HT$_{1B}$/ERG/SRP2070Fab (yellow), 5HT$_{2B}$/ERG (magenta), and β_2AR (blue), respectively.

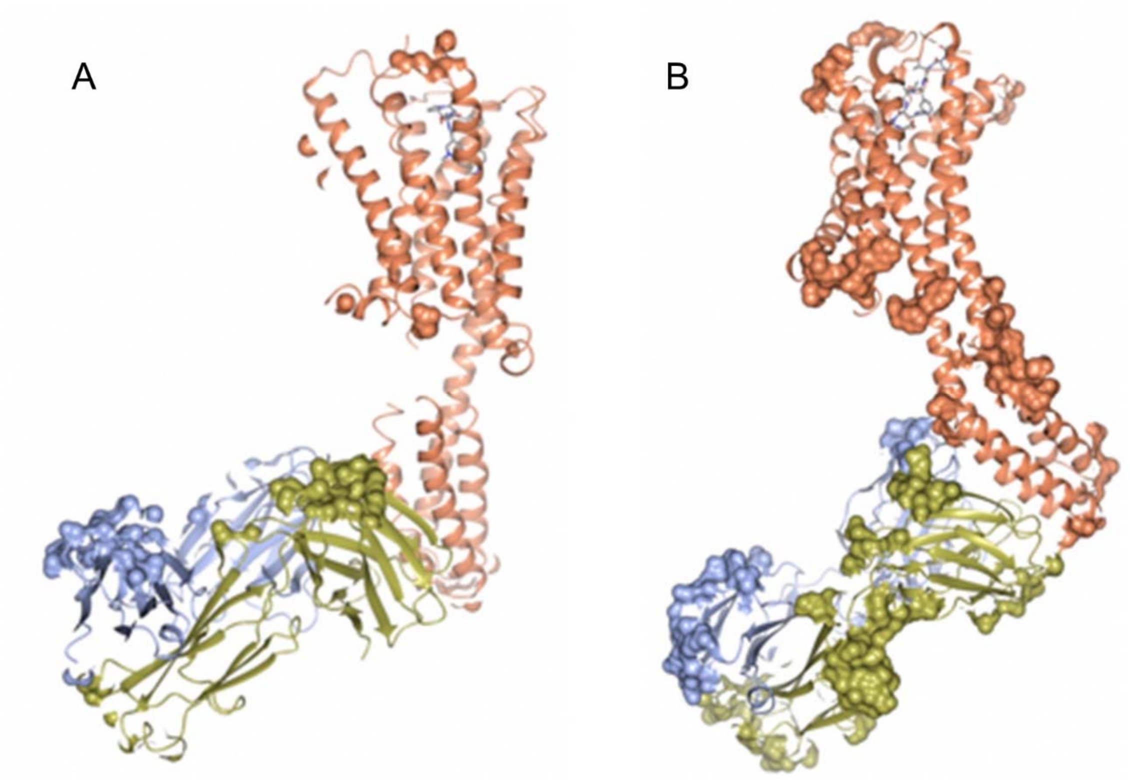

Supplementary Figure 4S: Analysis of crystal packing contact areas

(**A**) Structure of $5HT_{1B}$-BRIL/ERG/SRP2070Fab, $5HT_{1B}$ and BRIL (red), the light chain of SRP2070Fab (light purple), and the heavy chain of SRP2070Fab (yellow). The contact areas in the crystal packing are shown by the surface map. (**B**) Structure of AT_2R -BRIL/s-Ang II /SRP2070Fab, AT_2R and BRIL (red), the light chain of SRP2070Fab (light purple), and the heavy chain of SRP2070Fab (yellow). The contact areas in the crystal packing are shown by the surface map.

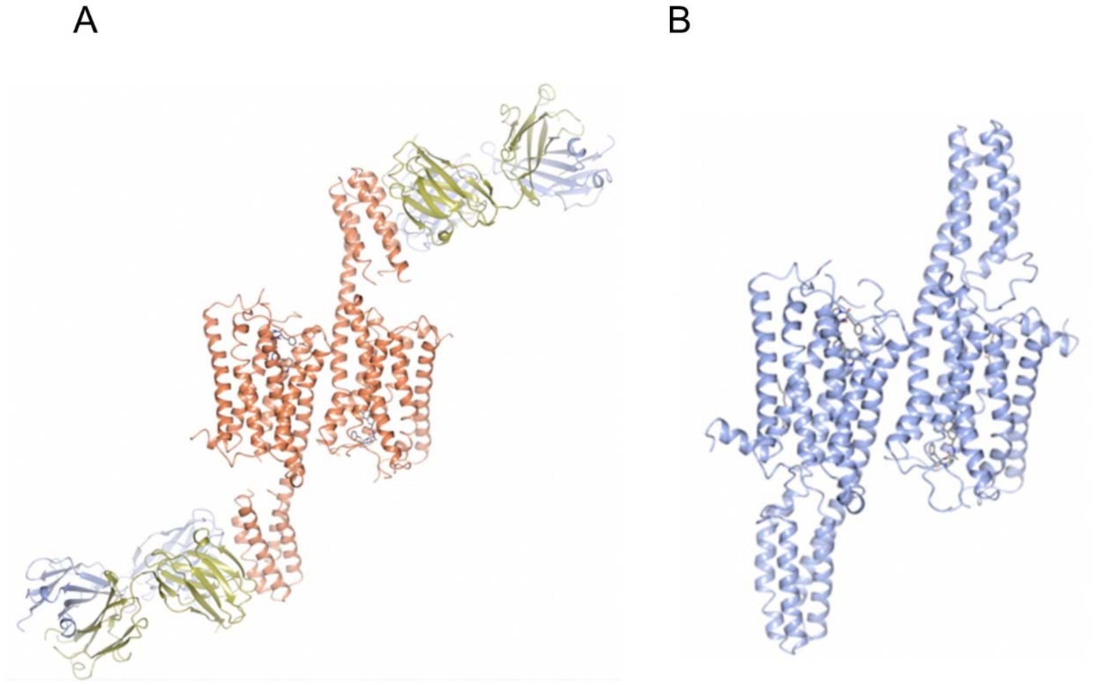

Supplementary Figure 5S: Structural comparison of 5HT$_{1B}$-BRIL/ERG/SRP2070Fab and 5HT$_{1B}$-BRIL/ERG (4IAR) in the crystal packing

(**A**) Structure of 5HT$_{1B}$-BRIL/ERG/SRP2070Fab, 5HT$_{1B}$ and BRIL (orange), L chain of SRP2070Fab (light purple), and H chain of SRP2070Fab (yellow). (**B**) Structure of 4IAR, 5HT$_{1B}$ and BRIL (light purple).
